# Effects of Lintnerization, Autoclaving, and Freeze-Thaw Treatments on Resistant Starch Formation and Functional Properties of Pathumthani 80 Rice Starch

**DOI:** 10.3390/foods8110558

**Published:** 2019-11-07

**Authors:** Sujitta Raungrusmee, Anil Kumar Anal

**Affiliations:** Department of Food, Agriculture and Bioresources, Asian Institute of Technology, Pathumthani 12120, Thailand; rsujitta@hotmail.com

**Keywords:** Phathumthani 80 (RD 31) rice, autoclaving, lintnerization, resistant starch, glycemic index

## Abstract

The objective of this study was to assess the effects of lintnerization, autoclaving, lintnerization followed by autoclaving, and freeze thawing treatments on the production of resistant starch from Pathumthani 80 (RD 31) rice. The produced resistant starch was further evaluated for some important physicochemical properties including pasting properties, swelling behavior, digestibility, water holding capacity, and functional properties including glycemic index and antioxidant properties. The lintnerization treatment and autoclaving significantly (*p* ˂ 0.05) increased resistant starch content to 64% (*w*/*w*) and gave the lowest glycemic index (46.12%). The lintnerization followed by autoclaving treatment significantly increased the solubility and water holding capacity, reduced the swelling power, and disrupted the crystalline structure of the starch granules. The native rice starch with autoclave treatment exhibited the highest swelling power among the samples, while the acid hydrolyzed starch was followed by autoclave treatment showing the lowest swelling power (1 g/g) at 90 °C. Fourier transform infrared analysis revealed the modified structures and bonding of the starch materials with the shifting of C=O stretch. However, the antioxidant properties and pasting properties were observed to decrease with the lintnerization, autoclaving, and freeze-thawing treatment of the native starch. The highly resistant starch content and low glycemic index value of the autoclaved RD 31 starch indicates the potential of the resistant starch’s application for the formulation of functional foods targeting the diabetic population.

## 1. Introduction

With the increase in consumer awareness toward healthier foods, there is a growing interest in functional foods with a low glycemic index. Glycemic index (GI) is defined as the increment in the blood glucose area following the test food and is expressed as the percentage of the corresponding area following a carbohydrate equivalent load of a reference product [1]. The glycemic index is a numeric value regarding which food is categorized into three groups. The three groups include high GI food (GI, 70 or high), medium GI food (GI, 56–69), and low GI food (GI, 55 or less). The glycemic index of rice varies with its varieties based mainly on the proportion of starch, particularly the ratio of amylose-amylopectin. A low glycemic index diet is usually comprised of high-amylose rice varieties. The intake of low GI foods has been observed to reduce the risk of coronary heart disease, diabetes, and obesity [2]. Resistant starch (RS) exhibits resistance to α-amylase activity and, hence, does not get digested in the upper gastrointestinal tract. Resistant starch is fermented by intestinal bacteria (probiotics) producing the short-chain fatty acids in the large intestine [3]. Due to its physiological health benefits and functional properties, resistant starch is gaining interest in the development of functional food products. Resistant starch plays an important role in preventing certain diseases including obesity and diabetes.

Based on physical and chemical attributes, resistant starch can be classified into five groups as RS 1 (physically inaccessible), RS 2 (raw starch granule), RS 3 (retrograded starch), RS 4 (chemically modified), and RS 5 (Amylose-lipid complex) [4]. Among all types of resistant starches, Resistant starch type 3 has greater commercial importance due to its high nutritional value and the crystalline polymorphs structure with a high endothermic transition (120–165 °C). Different treatment methods such as physical, chemical, and/or enzymatic methods are being utilized to modify native starch into the resistant starch [5]. Usually, native starch is modified into resistant starch type 3 by a combination of the gelatinization-retrogradation process. During the gelatinization step, heating of starch granules with excess water disrupts the starch granular structure, while, in retrogradation steps, amylose and amylopectin slowly recrystallize [6]. 

The degree of resistant starch formation is dependent on the nature of native starch and the treatments involved. Lintnerization or acid hydrolysis of α-1,4 and α-1,6 glycosides linkages from amylose and amylopectin develop resistance starch type 3 (lintnerized starch). Acid hydrolysis increases the crystalline content of starch, which, in turn, is resistant to enzymatic hydrolysis [7]. Several research studies have reported the formation of resistant starch type 3 by a repeated cycle of autoclaving-cooling treatment and freeze-thawing treatment. In a research study by Ratnaningsih [4], the resistant starch content and thermal stability of cowpea starch were observed to increase while GI decreased with the autoclaving-cooling treatment. Shrestha et al. [8] modified native green banana starch into resistant starch type 3 by lintnerization followed by autoclaving treatment. Furthermore, the lintnerized-autoclaved starch conjugated with soy protein was used as an encapsulant wall material to enhance thermal stability of astaxanthin, which can be utilized for biofortification of food and pharmaceutical formulations. In the freeze-thawing method, ice crystals are formed in starch gel during the freezing step while syneresis of water from the starch network occurs during the thawing step. These steps in combination lead to the formation of resistant starch type 3 [5]. The study of physicochemical and structural properties of waxy corn starch treated with repeated cycles of freeze-thaw treatments showed an increase in the number of pores on the starch surface and a decrease in starch crystallinity intensity [9].

Rice starch is a major source of nutrients and carbohydrates. Normally, rice starch can convert into glucose, which is the main energy source for metabolic function. During long-term overeating, carbohydrates potentially lead to some health problems such as type 2 diabetes, obesity, and colon diseases. Therefore, the development of starch resistant to enzymatic digestion from the native starch is recently gaining interest. The consumption of resistant starch is indicative of lowering the glycemic index. The starch digestion rate, the blood glucose level, and insulin responses of the most rice-based products are determined based on the amylose concentration. Amylose with its tightly packed structure exhibits high resistance to digestion compared to amylopectin [10]. The resistant starch type 3 is commercially prepared using high corn starch with high amylose content (˃40%). The Pathumthani 80 (RD 31) rice is native to Thailand and is classified as white rice with a hard texture, non-glutinous, aromatic, and with a high level of amylose [11]. 

The high amylose content of RD 31 is an important factor for determining its cooking and eating quality, hard texture, and its suitability for the development of resistant starch. The development of functional foods with the fortification of resistant starch has gained interest by food developers and nutritionists mainly due to fiber-fortification and the potential physiological benefits including the lowering of glycemic index [3]. The resistant starch has the potential to improve the functional properties of food including swelling behavior, solubility, digestibility, water binding capacities, and emulsion properties in food [9]. To the best of the authors’ knowledge, there are no reports yet published with the details of using different methods for developing resistant starch from rice, especially lintnerization, autoclaving, and lintnerization followed by autoclaving. Therefore, this study aims to develop the resistant starch from Pathumthani 80 (RD 31) paddy rice and to characterize some of its physicochemical and functional properties including in vitro glycemic index.

## 2. Materials and Methods 

### 2.1. Materials

Phatumthani 80 (RD 31), which is a type of a paddy rice, was provided by the Pathumthani Rice Research Center, Ministry of Agriculture and Cooperatives (MoAC), Thailand. The resistant starch test kit was bought from Megazyme International Ltd., Ireland. All the other chemical reagents used were of an analytical grade and bought from Sigma-Aldrich, Switzerland.

### 2.2. Extraction of Native Rice Starch

The rice flour (RF) was prepared by milling rice samples (RD 31) with a pin mill (Princess, Model No. 201994, Jiangsu, China) and sieved through a 100-mesh screen (Laboratory test sieve BS 410-1 Endecotts Ltd, London, England). The native rice starch (NRS) was extracted following the method described by Qin et al. [12] with slight modifications. Rice grains were soaked in sodium hydroxide (0.35% *w*/*v*, 1:2) at 4 °C for 24 h. The supernatant was drained, and the rice grains were ground with a blender (MX-AC400, Panasonic, Bangkok, Thailand) and passed through 100 mesh screens. The slurry was centrifuged (Beckman Coulter Centrifuge: Allegra X-15, Indianapolis, IN, USA) at 3000 rpm for 15 min. The supernatant was discarded, and the starch layer was resuspended with a triple volume of deionized water and centrifuged at 3000 rpm for 15 min. This process of washing the starch was repeated 4 times and the final pH was adjusted to 7 with hydrochloric acid (1 N). The starch slurry was further centrifuged at 3000 rpm for 15 min, which was followed by drying in a hot air oven (Binder, FD 260, Tuttlingen, Germany) at 60 °C for 24 h. The dried starch cake was ground by mean of grinder (Panasonic, MX-AC300, Osaka, Japan), which was followed by sieving through 100 meshes (Laboratory test sieve BS410-1 Endecotts Ltd, London, England), vacuum packed (Brother Packing Machinery: DZ400/2SBb, People’s Republic of China) in high-density polyethylene (HDP), and stored in a desiccator at 25 °C for further use. 

### 2.3. Preparation of Lintnerized Rice Starch

Lintnerization (acid hydrolysis) of the native rice starch sample was conducted by following the method, as described by Nasrin and Anal [6] with slight modifications. Sample starch was suspended into HCl solution at a different concentration (1 N, 1.5 N, and 2 N HCl solution) at 1:1.5 (*w*/*v*) ratios. The mixture of rice starch and HCl solution was warmed at 40 °C for 3 h. After adjusting the final pH to 6.5 using NaOH solution (10% *w*/*v*), the lintnerized starch was washed with distilled water and centrifuged at 1000 rpm for 5 min. The washed sample was dried in a hot air oven at 40 °C, cooled down, ground by using a grinder (Panasonic, MX-AC300, Osaka, Japan) into powder form, and passed through 100-mesh sieves (Laboratory test sieve BS410-1 Endecotts Ltd, London, England). The dried lintnerized starch was vacuum packed into the high-density polyethylene (HDP) pouch and stored in desiccators for further use.

### 2.4. Preparation of Autoclaved and Lintnerized-Autoclaved Rice Starch

Native rice starch and lintnerized rice samples (acid hydrolyzed at different concentrations) were suspended in water (1:10 *w*/*w*). The starch sample was gelatinized by heating in a water bath (Memmert: WNB 22, Buchenbach, Germany) maintained at 85 °C for 30 min. The pregelatinized starch sample was then autoclaved (Hirayama: HVE-50, Tokyo, Japan) at 135 °C for 30 min, cooled down, and stored at 4 °C for 24 h. This process of autoclaving and cold storing was repeated three times for each sample. The lintnerized-autoclaved starch was then dried in a hot air oven at 50 °C, cooled down, ground by using a grinder (Panasonic, MS-AC300, Osaka, Japan), sieved through 100 meshes (Laboratory test sieve BS410-1 Endecotts Ltd, London, England), vacuum packed in an HDP pouch, and stored in the desiccators. 

### 2.5. Preparation of Freeze-Thawed Rice Starch

Freeze-thawing of native starch was conducted by following the method described by Wang et al. [13] with some modifications. Native rice starch sample suspension in distilled water (10% *w*/*v*) was heated in boiling water for 30 min, which was followed by cooling down and freezing the samples at −20 °C for 24 h. The frozen sample was thawed in a water bath at 30 °C for 90 min and dried at 60 °C in a hot air oven until it was dry. The dried samples were milled by means of a grinder (Panasonic, MX-AC300, Osaka, Japan) and sieved through 100 meshes (Laboratory test sieve BS410-1 Endecotts Ltd, London, England), vacuum packed in HDP bags, and stored in a desiccator for a further experiment. 

### 2.6. Determination of the Amylose Content

Amylose content of the sample was determined by colorimetric measurement of the blue amylose iodine complex following the method of Juliano [14] with slight modifications. The rice sample (100 mg) was mixed with ethanol (1 mL) and 1 N sodium hydroxide (9 mL) and heated at 100 °C for 10 min. The mixture was allowed to cool, and the final volume was adjusted to 100 mL with distilled water. The starch solution (5 mL) was mixed with 1N acetic acid (1 mL) and iodine solution (2 mL). The final volume was adjusted to 100 mL. After 20 min of incubation, at 25 °C, the absorbance was measured with a spectrophotometer (Gene Quant 1300, Piscataway, NJ, USA) at 620 nm. The iodine reagent (1 mL) diluted to a final volume (50 mL) with distilled water was used as a blank. For the standard curve, potato amylose at different concentrations was treated the same way as the sample and the absorbance was plotted against the concentration. 

### 2.7. Determination of Resistant Starch, Non-Resistant Starch, and Total Starch

Resistant starch content of the sample was determined enzymatically using the Megazyme Resistant Starch assay procedure test kit (Megazyme International Ltd., Wicklow, Ireland). A sample (100 mg) with the mixture (4 mL) of pancreatic α-amylase (10 mg/mL) and amyloglucosidase (3 U/mL) was incubated in a shaking water bath at 37 °C for 16 h. Ethanol (99%, 4 mL) was added to the mixture to terminate the enzymatic reaction, which was followed by centrifugation (3000 rpm for 10 min). The supernatant was separated and used for determining non-resistant starch. The resistant starch in the pellet was mixed with ethanol (50%, 8 mL), stirred, and centrifuged (3000 rpm for 10 min), and the supernatant was decanted. The pellet was then mixed with KOH solution (2 M, 2 mL) with continuous stirring (300 rpm) in an ice water bath for 20 min followed by the addition of sodium acetate buffer (8 mL, pH 3.8) and amyloglucosidase (3300 U/mL, 0.1 mL). The mixture was incubated in a water bath (50 °C for 30 min) and centrifuged (3000 for 10 min). The supernatant (0.1 mL) was added to glucose oxidase-peroxidase-amino antipyrine (GOPOD) (3 mL) and incubated (50 °C for 20 min). The absorbance of the mixture was measured using spectrophotometer (Gene Quant 1300, Piscataway, NJ, USA) at 510 nm. The resistant starch content, non-resistant starch, and the total starch content were calculated using an equation from the kit manual. Total starches were calculated as the sum of resistant starch and non-resistant starch.

(1) $$\mathrm{Resistant}\;\mathrm{starch}={\Delta E}_{1}\;\times \;\frac{F}{W}\;\times 90$$

(2) $$\mathrm{Non}\text{-}\mathrm{resistant}\;\mathrm{starch}={\;\Delta E}_{2}\;\times \;\frac{F}{W}\;\times 90$$

(3) Total starch=Resistant starch +Non-resistant starch

where ΔE_1_ = absorbance of resistant starch sample - absorbance of blank, ΔE_2_ = absorbance of non-resistant starch sample - absorbance of blank, F = 100 (µg of D-glucose) divided by the glucose oxidase-peroxidase-amino antipyrine (GOPOD) absorbance for this 100 µg of D-glucose, W = dry weight of sample analyzed. 

### 2.8. Determination of Glycemic Index (GI)

The in-vitro glycemic index (GI) of the sample was determined following the method described by Goni et al. [15] with slight modifications. The glucose concentration was analyzed using a glucose oxidase-peroxidase kit (Megazyme International Ireland Ltd., Wicklow, Ireland), and the color reaction was measured in a Ultraviolet-isible (UV/VIS) spectrophotometer, at 510 nm. The glucose digestion rate was expressed as the percentage of glucose in each sample (mg glucose. 100 mg/sample) at each time interval (0, 30, 60, 90, 120, 150, and 180 min). Hydrolysis curves were developed, and the area under the hydrolysis curve (AUC) was calculated. The Hydrolysis Index (HI) for each sample was calculated as the ratio between the area under hydrolysis curve (AUC) of the sample and the reference sample (white bread) and expressed as a percentage. Lastly, the glycemic index was calculated, according to the equation below.

(4) GI=39.71 +(0.549 ×HI)

where GI = Glycemic Index (%) and HI = Hydrolysis Index (%).

### 2.9. Determination of Antioxidant Activity 

According to Sadiq et al. [16], antioxidant activity was analyzed by 2,2 diphenyl-1-picrylhydrazyl radical scavenging assay (DPPH) at 517 nm with a Ultraviolet/visible spectrophotometer. Sample aqueous extract (0.1 mL) was added to MeOH solution of 2,2 diphenyl-1-picrylhydrazyl radical scavenging assay (DPPH) (0.004%, 3 mL). After incubation (30 min) under dark, absorbance was measured at 517 nm and the percent inhibition activity was calculated using the following equation.

(5) $$\mathrm{Percent}\;\mathrm{inhibition}\;\left(\mathrm{mg}\;\mathrm{Trolox}/\;g\;\mathrm{sample}\right)=\;\left[\frac{A_{0}-A_{e}}{A_{0}}\;\times 100\right]$$

where A_o_ = absorbance without extract, A_e_ = absorbance with extract.

Ferric ion reducing antioxidant power assay (FRAP) was measured following Sadiq et al. [17] with a slight modification. The ferric ion reducing antioxidant power (FRAP) reagent (1.5 mL) and sample extract (50 µL) were mixed and incubated at 37 °C for 4 min. Absorbance was measured at 593 nm with a UV-visible spectrophotometer and expressed as mg FeSO4 per 100 g sample.

Total phenolic content was analyzed following Folin and Ciocalteu [18] using a UV-visible spectrophotometer at 760 nm. The report was expressed as mg gallic acid (Sigma-Aldrich, Switzerland) equivalent (GAE)/100 g sample.

### 2.10. Determination of Pasting Properties

The pasting properties of starch samples were analyzed by a Rapid Visco Analyzer (RVA; Model 4, Newsport Scientific, New South Wales, Australia). A sample (2.5 g) was mixed uniformly with distilled water (25 mL). The sample suspension was subjected to four settings including preheating at 50 °C for one min, heating until temperature rise to 95 °C, holding at 95 °C for 3.2 min, and cooling to 50 °C. Samples were mixed and homogenized at 960 rpm for 10 s at the beginning of the test, and then speed was reduced to 160 rpm and continued throughout the test. The total analysis time was 13 min. Peak viscosity, trough, breakdown viscosity, final viscosity, setback viscosity, peak time, and pasting temperature were obtained by the rapid visco analyzer (RVA).

### 2.11. Determination of Swelling Power and Solubility 

The swelling power and solubility of the tested samples were calculated following Shrestha et al., [8] with slight modifications. The rice sample (1 g) dispersed in distilled water (50 mL) in a centrifuge tube was heated into the water bath at different temperatures (60–95 °C) for 30 min with continuous stirring. After cooling to room temperature, the supernatant was decanted and dried (105 °C about 5 h), and the weight of the sediment was noted. The swelling power and solubility were calculated using the following equations. 

(6) $$\mathrm{Swelling}\;\mathrm{power}\;\left(g/g\right)=\frac{\mathrm{weight}\;\mathrm{of}\;\mathrm{wet}\;\mathrm{residue}}{\mathrm{weight}\;\mathrm{of}\;\mathrm{dry}\;\mathrm{residue}\;}\;\times 100$$

(7) $$\mathrm{Solubility}\;\left(\%\right)=\;\frac{\mathrm{weight}\;\mathrm{of}\;\mathrm{dry}\;\mathrm{sample}\;\mathrm{in}\;\mathrm{supernatant}\;}{\mathrm{weight}\;\mathrm{of}\;\mathrm{dry}\;\mathrm{sample}\;}\;\times 100.\;$$

### 2.12. Determination of Water Holding Capacity (WHC) 

The water holding capacity of the sample was determined, according to the method of Rodriguez-Ambriz et al. [19] with slight modifications. The sample (1 g) was dispersed in distilled water (50 mL) into the centrifuge tube and heated in a water bath at different temperatures (40–90 °C) for 30 min with continuous stirring. After cooling to room temperature, the tube was centrifuged at 3000 rpm for 20 min. The supernatant was discarded, the sediment was weighed and dried, and the water holding capacity was determined according to the following formula.

(8) $$\mathrm{WHC}\;\left(g/g\right)=\frac{\mathrm{weight}\;\mathrm{of}\;\mathrm{wet}\;\mathrm{residue}-\mathrm{weight}\;\mathrm{of}\;\mathrm{dry}\;\mathrm{residue}}{\mathrm{weight}\;\mathrm{of}\;\mathrm{dry}\;\mathrm{residue}\;}$$

### 2.13. Scanning Electron Microscopy

The microstructural images of the sample were taken using a Scanning Electron Microscope (SEM) (JSM 6310F, Tokyo, Japan). The sample particles were sprayed over the adhesive carbon tape fixed with a circular brass stub and placed in a vacuum chamber. After reaching a vacuum of 8 Pa and plasma current of 15 mA, the gold particles were splattered for 90 s. The stub with gold splattered samples was then kept inside the Scanning Electron Microscope (SEM) chamber for imaging. The images were analyzed at 6000× resolution.

### 2.14. Chemical Finger Printing of Produced Starch by Fourier Transform Infrared (FTIR) Spectroscopy

The infrared spectra of the sample were measured using an Attenuated Total Reflectance (ATR) FTIR (Alpha-E: BRUKER, Ettlingen, Germany) with a spectra-Tech HATR accessory. The sample was used directly for measuring the spectrum from 500 to 4000 cm^−1^ wave numbers. Each spectrum was the average of eight scans. All measurements were conducted under ambient conditions.

### 2.15. Color

Color spectra of the sample were determined by using a Hunter-Lab spectrophotometer (Color Flex: 45/0, Reston, VA, USA). The sample (10 g) was placed in the glass container and put over the slit of the instrument. The average value of 10 measurements was reported and showed as L* (lightness), a* (redness), and b* (yellowness) values. The color intensity (B) was calculated by following Gavahian et al. [20] and using the equation below.

(9) $$B=\;\sqrt{{\left(a*\right)}^{2}+{\left(b*\right)}^{2}}$$

### 2.16. Statistical Analysis

All the experiments were conducted in triplicate and means ± standard deviations were reported. The analysis of variance was performed by one-way ANOVA procedures of MSTAT-C software (Statistical Package developed by the Michigan State University, East Lansing, MI, USA). Comparisons among the samples were further analyzed by Tukey’s honestly significant difference (HSD) test at a 95% confidence level. 

## 3. Results and Discussion

### 3.1. Chemical Composition 

The chemical composition of RD 31 rice flour, native rice starch, and modified starch were investigated (Table 1). The total starch of native rice starch (96.05 ± 0.72%) was higher than the rice flour (86.81 ± 0.70%). The lower total starch content of rice flour compared to rice starch might be due to the presence of some outer layers, namely pericarp, Testa, the nucellus, and the aleurone layer [21]. On the other hand, alkaline treatment involved during the starch extraction helped to separate starch from the binding protein, and other matters improve the purity of starch products [22]. The RD 31 is categorized as hard non-glutinous aromatic rice with high amylose content. Based on amylose content, rice is classified as waxy (0–2% amylose), low (10–20% amylose), intermediate (20–25% amylose), and high (more than 25% amylose) [23]. The amylose content of native rice starch (45.17 ± 1.54%) was found to be significantly higher than the native rice flour (33.92 ± 0.15%). 

The amylose content of the lintnerized or acid hydrolyzed starch (35.57 ± 0.73 to 41.48 ± 0.48%) and lintnerized. This was followed by autoclaved starch (21.70 ± 0.66% to 28.96 ± 0.26), which was observed to decrease significantly. The earlier reports also suggested the decrease in amylose content of starch, including the decrease in amylose content of native culled banana starch from 39.88% to 34.05% [3]. A decrease occurred in the amylose content of corn starch from 16.9% to 13.3% [24] with acid hydrolysis. This decrease in the amylose content during acid hydrolysis is due to the action of acid on the amorphous regions of the starch where amylose resides [25]. 

However, the amylose content of starch resulting from autoclaving (45.97 ± 0.79%) and freeze thaw treatment (43.59 ± 0.34%) was significantly similar to the native starch. During autoclaving treatment, starch was gelatinized at a temperature of 135 °C under pressure whereby the starch granules become fully disrupted. Upon cooling, the amylose chains can associate to form hydrogen bond stabilized double helices [26]. Moreover, the freeze thaw treatment resulted in a high degree of syneresis in gelatinized starches and accelerated the retrogradation. Amylose content is often used to predict the starch digestion rate, blood glucose, and insulin responses to rice. Starchy foods that are rich in amylose content are associated with lower blood glucose levels and slower emptying of the human gastrointestinal tract compared to those with low levels of amylose [27].

The formation of resistant starch is influenced by several factors, including amylose content, molecule chain length, autoclaving temperature, storage time, and starch gel temperature [5]. The resistant starch content of native rice starch (8.44 ± 0.51%) was significantly higher than that of the rice flour (3.84 ± 1.01%). The resistant content was observed to increase significantly (*p* < 0.05) with the lintnerization treatment so that resistant starch content increased with a rise in the concentration of hydrochloric acid starting from 1 N (13.73 ± 0.48%), 2 N (22.99 ± 0.48%), and 3 N (40.19 ± 2.96%). This increase in resistant starch content with the lintnerization treatment might be due to the disruption of amorphous regions by the acid leading to an increase in the ratio of crystalline parts, which are more difficult for enzymes to access [28]. 

The resistant starch content was further observed to increase significantly with the lintnerization method, which was followed by the autoclaving treatment. However, the highest value of resistant starch was exhibited by the autoclaved starch (64.95 ± 0.26%). During autoclaving treatment, the starch granules become fully disrupted, which, upon cooling, form double helices stabilized by hydrogen bonds. Thus, the repeated autoclaving-cooling treatments lead to the formation of resistant starch type 3 crystallites, which are resistant to starch hydrolyzing enzymes due to their tightly packed structure [3]. The formation of resistant starch is strongly related with the amylose content since the formation of resistant starch involves the crystallization of amylose [29]. The crystallization of amylose is expected to reduce available α-glucan chains to which α-amylase can bind, which, in turn, reduces the susceptibility of retrograded starch to digestion [30]. Resistant starch formation is further dependent on the repeated cycle of autoclaving and retrogradation treatment such that increasing the number of cycles to 20 raised the resistant starch level to more than 40% [31]. On the other hand, compared to native starch, the resistant starch slightly increased in the freeze-thaw starch, which might be due to the linear structure of amylose in the gelatinized starch with a great tendency to form double helices [3].

In this work, analysis of the data trends indicate that more resistant starch could be generated at the higher concentrations of HCl. The lintnerization followed by autoclaving starch had higher resistant starch content than the starch treated by lintnerization alone. As shown in Table 1, among all treatments, autoclaved RD 31 had the highest resistant starch, which indicates that the autoclaving treatment has a high impact on increasing the resistant starch. With the increase in resistant starch content, a significant decrease in the glycemic index was also observed (Table 2). Therefore, the autoclaving treatment seems to be suitable for the foods that require relatively high solubility and high-water holding capacity.

### 3.2. Glycemic Index 

The in vitro glycemic index (GI) of the rice flour, native starch, and the treated starch was investigated (Table 2). The GI value of rice flour (61.10 ± 0.02%) was significantly (*p* < 0.05) lower when compared to the native starch (66.32 ± 0.04%). Furthermore, compared with lintnerized rice starch (51.94 ± 0.01–58.94 ± 0.07%), the autoclaved rice starch showed a significantly lower glycemic index value (46.12 ± 0.01 to 56.83 ± 0.07%). All samples, except the starch treated with 1 N hydrochloric acid and the freeze thawed one, could be categorized as low glycemic index starch. The lower GI value of rice flour is due to the presence of various bioactive compounds including dietary fiber, resistant starch, and oligosaccharides. The presence of dietary fiber has been associated with a decrease in glucose, insulin, and serum lipid concentrations in both diabetic and non-diabetic persons [32]. 

The autoclaved starch significantly (*p* < 0.05) exhibited the highest resistant starch and the lowest glycemic index. In relation to resistant starch content, the higher the resistant starch content is, the slower the digestion of rice and the lower the glycemic index is. Resistant starch decreases postprandial glucose and insulin responses and, hence, decreases the glycemic index of food [33]. Considering the amylose content and glycemic index effects, the starchy foods with high amylose levels are associated with lower blood glucose levels and slower emptying of the human gastrointestinal tract. Therefore, such conditions are relevant, especially in the formulation of diets for diabetics [34].

### 3.3. Antioxidant Properties

The antioxidant properties of rice flour, native starch, and the treated starch were evaluated and expressed in terms of total phenolic content (TPC), 2,2 diphenyl-1-picrylhydrazyl (DPPH) radical scavenging activity, and the FRAP (Ferric ion reducing antioxidant power) ssay (Table 3). The antioxidant properties of the rice sample are due to the naturally occurring flavonoid compound present in the bran layer of rice. Consequently, the untreated rice flour had the highest antioxidant capacity (100.03 ± 1.92 mg GAE/100 g, 38.75 ± 0.04 mg Trolox/100 g sample, and 192.17 ± 0.54 FeSO_4_/100 g) due to the presence of the phenolic compounds in the aleurone layer and the pericarp layer of the rice kernel. 

However, immediately after rice starch extraction, the antioxidant properties reduced significantly (*p* < 0.05), which might be due to removal of the rice bran layer. Additionally, the acid hydrolysis, autoclaving, and freeze-thawing treatments caused a significant decrease in the total phenolic compounds as well as in antioxidant activity. Fengmei [35] reported the reduction of antioxidant activity of oranges by 50% after the hydrochloric acid treatment. Moreover, a significant difference (*p* < 0.05) in TPC, DPPH, and FRAP was observed between lintnerization, which was followed by autoclaving treatment and freeze-thaw treatment with the lowest value in lintnerized-autoclaved starch samples. Several research studies have reported that free phenolic compounds are more susceptible to loss due to hydrothermal treatment and heat treatment [36]. 

### 3.4. Pasting Properties 

The viscosity and pasting properties of rice flour, native starch, and the treated starch were analyzed by a rapid viscometer analyzer (Table 4). The highest viscosity values were exhibited by the rice flour while all the viscosity values were observed to decrease with acid hydrolysis and autoclave treatment. Moreover, the viscosity of the acid hydrolyzed starch sample decreased with the increase of the acid concentration, which might be due to the cleavage of the starch chain leading to a decrease in the molar mass of the starch [37]. For the autoclaved starch, break down and final viscosity of the starch were significantly lower than in the native, lintnerized, and freeze thawed starch. 

The pasting property is dependent on several factors including the amylose and amylopectin chain length, the leaching of amylose, the starch crystallinity, etc. [38]. A negative correlation between viscosity and amylose content was observed by Kaur et al. [39]. The autoclaved starch showed the lowest break down viscosity due to the amount and the molecular weight of amylose leached from the granules and the remnant of the gelatinized starch [40]. Furthermore, the acid treated sample had a lower setback compared with the rice flour and native rice starch, which indicates its tendency toward retrogradation. Nevertheless, the freeze-thaw treatment slightly increased the viscosity properties of starch. Starch subjected to hydrothermal treatment involving freezing–thawing procedures is characterized by increased viscosity [41].

### 3.5. Solubility, Swelling Power, and Water Holding Capacity 

**Figure 1:**
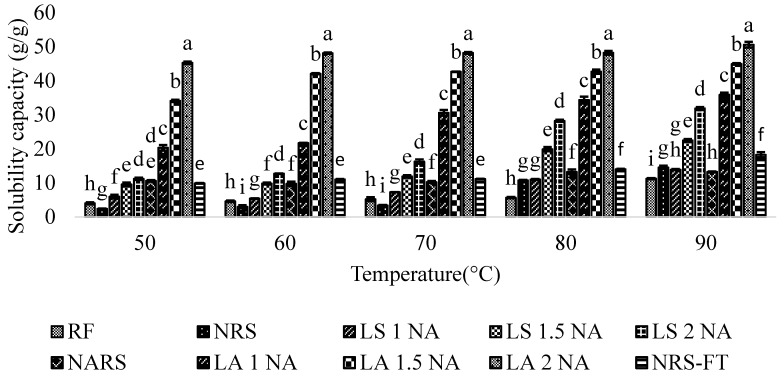
Solubility capacity of the RD 31 flour, native starch, and modified starches at different temperatures. Different suffix (a–f) within the same temperature indicate significant differences (*p* < 0.05). RF = Rice flour. NRS = Native rice starch. NARS = Native autoclaved resistant starch LS 1 NA = Lintnerized starch treated with 1 N HCl. LS 1.5 NA = Lintnerized starch treated with 1.5 N HCl. LS 2 NA = Lintnerized starch treated with 2 N HCl. LA 1 NA = Lintnerized starch treated with 1 N HCl and autoclaved. LA 1.5 NA = Lintnerized starch treated with 1.5 N HCl and autoclaved. LA 2 NA = Lintnerized starch treated with 2 N HCl and autoclaved. NRS - FT = Native rice starch-Freeze thawed.

The solubility of the starches increased with the increase in the temperature (Figure 1). This increase in solubility is due to the growth in the mobility of the starch granules, which facilitated the dispersion of starch molecules in water. Compared to native starch, solubility values of the lintnerized starch was observed to increase with the increase in the temperature. This rise in solubility of lintnerized starch might be due to the greater starch chain depolymerization induced by the prolonged acid treatment [42]. Moreover, the modified starch with acid hydrolysis combined with autoclaving treatment exhibited the highest solubility capacity at all temperatures. This increase in solubility capacity of lintnerized-autoclaved starch is attributed to change in granular structure, reduction in molecular weight, and increase in amylose content. Research studies by Koksel [43] and Ozturk [44] have reported a significant increase in water solubility capacity and water binding capacity as a result of heating and autoclaving treatments. This increase in solubility capacity of treated starch indicates that the lintnerization-autoclaving treatment method for the preparation of resistant starch is suitable for the development of food products requiring high water binding properties [5]. 

**Figure 2:**
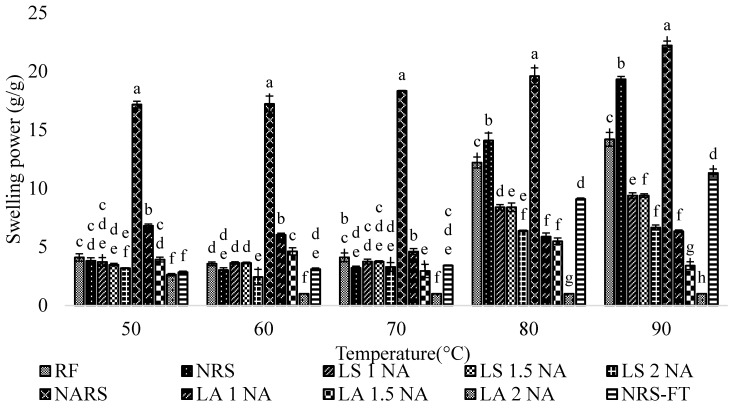
Swelling power of the Pathumthani 80 (RD 31) rice flour, native starch, and modified starches at a different temperature. Different suffix (a–h) within the same temperature indicate significant differences (*p* ˂ 0.05). RF = Rice flour. NRS = Native rice starch. NARS = Native autoclaved resistant starch. LS 1 NA = Lintnerized starch treated with 1 N HCl. LS 1.5 NA = Lintnerized starch treated with 1.5 N HCl. LS 2 NA = Lintnerized starch treated with 2 N HCl. LA 1 NA = Lintnerized starch treated with 1 N HCl and autoclaved. LA 1.5 NA = Lintnerized starch treated with 1.5 N HCl and autoclaved. LA 2 NA = Lintnerized starch treated with 2 N HCl and autoclaved. NRS-FT = Native rice starch-Freeze thawed.

Swelling power is determined after heating the starch in excess water and is expressed as the ratio of the wet weight of the (sediment) gel formed to its dry weight. It depends on the processing conditions (temperature, time, stirring, and centrifugation) and is thought of as its water binding capacity. The native rice starch with autoclave treatment (NARS) exhibited the highest swelling power among the samples (19.32 ± 1.06 g/g), while the acid hydrolyzed starch (2 N HCl), which was followed by autoclave treatment (LA 2 NA) showing the lowest swelling power (1.00 ± 0.00 g/g) at 90 °C (Figure 2). Swelling power of starch is greatly influenced by the amylopectin content and the amylose/amylopectin distribution. The destruction of the starch granular structure during acid hydrolysis might be responsible for the lower swelling power of lintnerized-autoclaved starch. The acid hydrolysis results in leaching of the amylose and in increasing short chain amylopectin results in a branched structure with less ability to swell [45].

**Figure 3:**
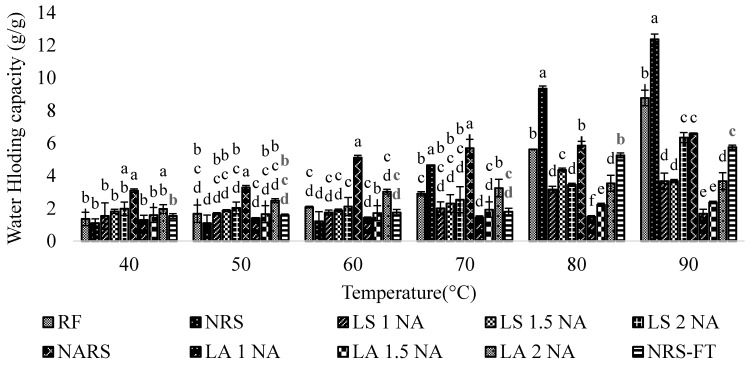
Water holding capacity of the RD 31 rice flour, native starches, and modified starches at different temperatures. Different suffix (a–f) within same temperature indicate significant differences (*p* < 0.05). RF = Rice flour. NRS = Native rice starch. NARS = Native autoclaved resistant starch. LS 1 NA = Lintnerized starch treated with 1 N HCl. LS 1.5 NA = Lintnerized starch treated with 1.5 N HCl. LS 2 NA = Lintnerized starch treated with 2 N HCl. LA 1 NA = Lintnerized starch treated with 1 N HCl and autoclaved. LA 1.5 NA = Lintnerized starch treated with 1.5 N HCl and autoclaved. LA 2 NA = Lintnerized starch treated with 2 N HCl and autoclaved. NRS - FT = Native rice starch-Freeze thawed.

Water holding capacity is greatly influenced by the physical conditions of the starch molecule including dietary fiber, protein, and amylose content [19]. The water binding capacity of native and modified starches submitted to heating from 40 °C to 90 °C was analyzed (Figure 3). With the increased temperature up to 90 °C, water binding capacity showed a tendency to increase. The water holding capacity of the RD 31 flour and native starch was observed to increase up to 8.77 ± 0.48 and 12.37 ± 0.32 g/g, respectively, at 90 °C, while the lintnerized starch exhibited the lower water holding capacity as compared to that of RD 31 rice flour and native starch. Low water holding capacity of acid treated starch could be attributed to reducing the amorphous region in the starch granule, which, in turn, reduces the number of available binding sites for water in starch granules [46]. However, the water holding capacity was observed to increase with the rise in acid concentration, which might be due to the rise in the low molecular weight starch with hydroxyl groups that can hold water molecules [47]. Furthermore, the water holding capacity of native autoclaved starch was higher than lintnerized starch, which might have resulted from the linear chains produced by breakage of amylopectin branches [5].

### 3.6. Morphology of Starch Granules

**Figure 4:**
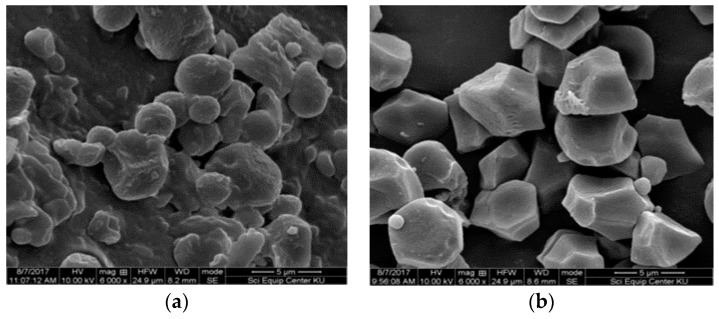

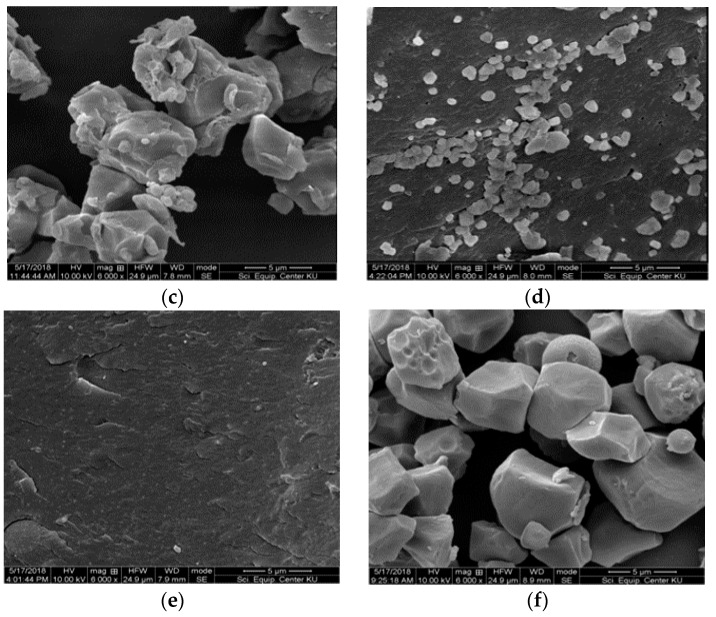
Morphology of starch as measured by scanning electron microscopy at 6000X. (**a**) Rice flour. (**b**) Native rice starch. (**c**) Starch treated with 2 N HCl. (**d**) Starch treated with 2 N HCl and autoclaved. (**e**) Native rice starch: autoclaved. (**f**) Native rice starch: freeze thawed.

Rice starch granules are the smallest among the other cereal grains starch and exit as discrete particles. The morphology of RD 31 rice flour, native rice starch, and all the treated starches were photographed under a scanning electron microscope (SEM) at a magnification of 6000X (Figure 4). The surface of rice flour appeared to be rough with lost flatness and smoothness (Figure 4a). Meanwhile, native starch granules had a smooth surface with an angular, polygonal, and irregular shape (Figure 4b).

The shape and size of starch granule were observed to change with acid hydrolysis and heat treatment. The morphology of acid hydrolyzed starch appeared to be rougher than the native starch, which indicated that acid treatment caused the erosion of the core part of the starch granules (Figure 4d). This difference in morphology of acid treated and native starch signifies the action of acid in relation to structural and functional properties of the starch [48]. The scanning electron microscope (SEM) image of the autoclaved starch clearly showed the disappearance of the granular structure of native starch and the formation of stone such as irregularly shaped granules (Figure 4f). This morphological change of autoclaved starch might be due to the aggregation of linear starch fragments and retrogradation occurring during the repeated cycles of autoclaving and cooling [40]. With one cycle of autoclaving and cooling, the morphology of freeze thawed-starch granules appeared to be similar to native starch with no noticeable difference (Figure 4f). Wang et al. [49] has reported the collapse of the network structure of starch with a regular arrangement of starch molecules due to the repeated freeze thaw treatment.

### 3.7. FTIR Analysis

**Figure 5:**
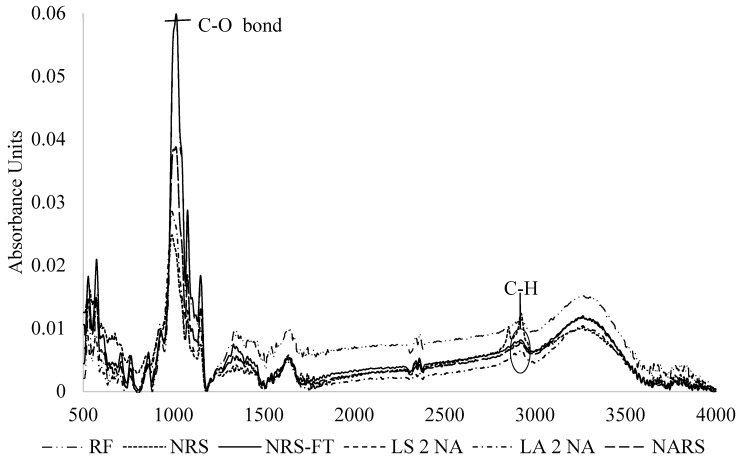
**Fourier Transform Infrared** (FTIR) spectra of the RD 31 rice flour, native starch, and modified starches. RF = Rice flour. NRS = Native rice starch. NRS – FT = Native rice starch: freeze thawed. NARS = Native autoclaved resistant starch. LS 2 NA = Lintnerized starch treated with 2 N HCl. LA 2 NA = Lintnerized starch treated with 2 N HCl and autoclaved.

The changes in chemical finger printing of native starch with acid hydrolysis, autoclaving, and freeze-thaw treatments were analyzed by the Fourier Transform Infrared (FTIR) spectrum (Figure 5) and the functional groups were assigned to the corresponding peaks in the region of 500 to 4000 cm^−1^. The peak intensity and shape of lintnerized factors. Lintnerization was followed by autoclaving. Autoclaved and freeze thawed RD 31 rice starches were significantly different than that of rice flour and native rice starch. The change in peak intensity and shape, therefore, indicates the modification in the starch granular structure and the bonding of the starch substrates with the different treatment.

In all spectra, a composite variation mode in the region below 800 cm^−1^ is related to the pyranose ring in the glucose unit. The anhydrous glucose ring O=C stretching is represented by the peak between 990 cm^−1^ and 1030 cm^−1^ [6]. The OH-absorption of the RD 31 starch was observed at approximately 3000–3500 cm^−1^. The intensities of the peak of the acid hydrolyzed starch are the feature peaks of the C-O-C and C-O bond of the glycosidic linkage in the starch [50]. Nevertheless, the RD 31 starch can be used to investigate the C-H stretching region at approximately 2800-3000 cm^−1^. The most intense peak of glucose is characteristic of the C-O stretch vibration [51]. The sifting and mode of peak changes depend on the stability and strength of hydrogen bonds. Compared to native starch, the autoclaved starch showed higher intensity at 1000 cm^−1^, which were similar to findings of Ashwar et al. [52] who reported an increase in Fourier Transform Infrared (FTIR) intensity at 1047 cm^−1^ in autoclaved rice starch. This increased intensity indicates that the autoclaving treatment causes more efficient packing of double helices within the crystalline lamella. Similarly, a research study by Basilio-Cortés et al. [53] showed the enhanced band at 1660 cm^−1^ in the spectra of modified corn starch, which signifies the changes in the starch crystallinity resulting from an interaction of hydrochloric acid-starch-succinic anhydride. 

### 3.8. Color Value 

The color value of RD 31 rice flour, native rice starch, and modified starch were studied by the colorimeter and expressed in terms of the CIE L*a*b* color system (Table 5). The native rice starch showed a higher L value and lower a and b values compared to rice flour due to the native starch appearing to be brighter than the rice flour. The presence of bran layer, typically composed of aleurone and sub-aleurone layer, in the rice flour is responsible for the color of the rice kernel. The bran layer present in rice flour is removed during the alkaline native starch extraction process. The color intensity of the native rice starch was observed to increase significantly with the increase in the concentration of HCl during the lintnerization treatment. The native autoclaved resistant starch and lintnerized (2 N) autoclaved resistant starch showed the highest color intensity, which indicates the darker color of the modified starch.

**Figure 6:**
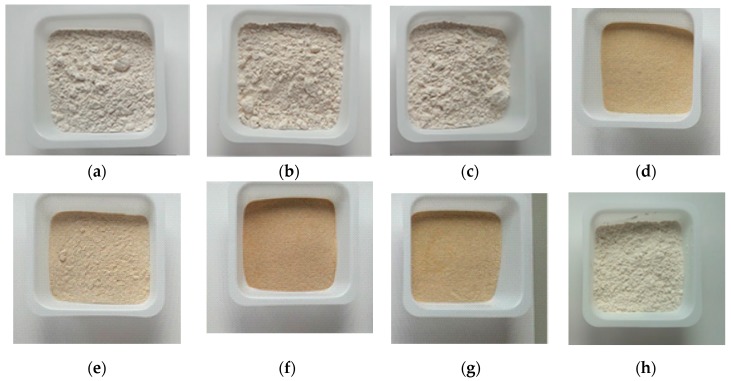
Photographic image of acid hydrolyzed, autoclaved, and freeze thawed starch (**a**) Starch treated with 1 N HCl. (**b**) Starch treated with 1.5 N HCl. (**c**) Starch treated with 2 N HCl. (**d**) Native rice starch-autoclaved rice flour. (**e**) Starch treated with 1 N HCl and autoclaved. (**f**) Starch treated with 1.5 N HCl and autoclaved. (**g**) Starch treated with 2 N HCl and autoclaved. (**h**) Native rice starch: freeze thawed.

The “L” value was observed to decrease, while “a*” value and “b*” value increased significantly (*p* ˂ 0.05) with the lintnerization treatment and lintnerization followed by the autoclaving treatment with respect to the native starch. In comparison to non-autoclaved counterpart, lintnerization followed by autoclaving treatment starch appeared to be darker in color (Figure 6). The effect of an acid can lead to smaller particle sizes. Furthermore, the darker color of autoclaved starch was due to non-enzymatic browning of the starch caused by the Maillard reaction between reducing sugars from the heated starch and the amino group in the proteins during modification [54]. However, the “L”, “a*” and “b*” color parameters of the freeze-thawed starch had no significant (*p* ≥ 0.05) differences in comparison with the native starch.

## 4. Conclusions

This study revealed that dual autoclaving and the lintnerization treatment can cause the reorganization of the amylose and amylopectin chains of starch, which significantly improved its physicochemical characteristics and digestibility, and, thereby, lowered glycemic properties. The Pathuthani 80 (RD 31) rice cooked with autoclaving gave the highest resistant starch (64.95% *w*/*w*) with the lowest glycemic index (46.12%), which was followed by lintnerized-autoclaved starch. The viscosity of the native rice starch was observed to decrease with lintnerization and autoclaving treatment. The acid hydrolyzed and autoclaved starch showed the highest solubility tendency and swelling power with reduced water holding capacity. The FTIR analysis proved that autoclaving and lintnerization treatments modified the native rice starch at a molecular level. The surface of the modified starch by lintnerization appeared to be rougher, whereas more compact surfaces were observed with the autoclaved starch. The results of this study indicate the potential of the application of the lintnerized and autoclaved Pathumthani 80 rice starch as a source of resistant starch for developing functional foods with a low glycemic value. 

## Figures and Tables

**Table 1:** Chemical composition of the Pathumthani 80 (RD 31) rice flour, native starch, and modified starches.

Type of Starch	Amylose Content (%)	Resistant Starch (%)	Non-Resistant Starch (%)	Total Starch (%)
RF	33.92 ± 0.15 ^e^	3.84 ± 1.01 ^j^	74.96 ± 2.67 ^bc^	86.81 ± 0.70 ^cd^
NRS	45.17 ± 1.54 ^ab^	8.44 ± 0.51 ^i^	87.62 ± 0.20 ^a^	96.05 ± 0.72 ^bcd^
LS 1 NA	41.48 ± 0.48 ^c^	13.73 ± 0.48 ^h^	83.92 ± 2.67 ^a^	97.65 ± 0.31 ^ab^
LS 1.5 NA	35.57 ± 0.73 ^de^	22.99 ± 0.48 ^f^	70.66 ± 2.23 ^c^	93.65 ± 0.13 ^d^
LS 2 NA	36.48 ± 0.55 ^d^	40.19 ± 2.96 ^d^	56.94 ± 1.58 ^d^	97.12 ± 1.38 ^abc^
NARS	45.97 ± 0.79 ^a^	64.95 ± 0.26 ^a^	32.31 ± 0.98 ^g^	97.26 ± 0.62 ^abc^
LA 1 NA	28.96 ± 0.26 ^f^	35.70 ± 0.56 ^e^	58.82 ± 0.75 ^d^	94.51 ± 0.24 ^cd^
LA 1.5 NA	25.31 ±0.59 ^g^	49.08 ± 0.04 ^c^	50.52 ± 0.24 ^e^	99.60 ± 0.27 ^a^
LA 2 NA	21.70 ± 0.66 ^h^	54.95 ± 0.60 ^b^	44.65 ± 0.28 ^f^	99.60 ± 0.34 ^a^
NRS - FT	43.59 ± 0.34 ^b^	18.00 ± 0.30 ^g^	77.49 ± 1.48 ^b^	95.49 ± 0.17 ^bcd^

Results expressed as mean of triplicate determinations ± standard deviation. Different superscripts within one column denote statistically significant differences (*p* < 0.05). RF = Rice flour. NRS = Native rice starch. NARS = Native autoclaved resistant starch. LS 1 NA = Lintnerized starch treated with 1 N HCl. LS 1.5 NA = Lintnerized starch treated with 1.5 N HCl. LS 2 NA = Lintnerized starch treated with 2 N HCl. LA 1 NA = Lintnerized starch treated with 1 N HCl and autoclaved. LA 1.5 NA = Lintnerized starch treated with 1.5 N HCl and autoclaved. LA 2 NA = Lintnerized starch treated with 2 N HCl and autoclaved. NRS - FT = Native rice starch-Freeze thawed.

**Table 2:** Hydrolysis index (HI) and glycemic index (GI) of the Pathumthani 80 (RD 31) rice flour, native starch, and modified starches.

Type of Starch	HI	GI
RF	38.98 ± 0.04 ^b^	61.10 ± 0.02 ^b^
NRS	48.48 ± 0.06 ^a^	66.32 ± 0.04 ^a^
LS 1 NA	35.03 ± 0.13 ^d^	58.94 ± 0.07 ^d^
LS 1.5 NA	27.60 ± 0.22 ^f^	54.86 ± 0.11 ^f^
LS 2 NA	22.29 ± 0.01 ^g^	51.94 ± 0.01 ^g^
NARS	11.68 ± 0.04 ^i^	46.12 ± 0.01 ^i^
LA 1 NA	30.91 ± 0.49 ^e^	56.83 ± 0.07 ^e^
LA 1.5 NA	12.50 ± 0.40 ^h^	46.57 ± 0.22 ^h^
LA 2 NA	12.21 ± 0.17 ^h^	46.41 ± 0.09 ^hi^
NRS - FT	37.36 ± 0.27 ^c^	60.22±0.14 ^c^

Results expressed as mean of triplicate determinations ± standard derivation. Different superscripts within one column denote statistically significant differences (*p* < 0.05). RF = Rice flour. NRS = Native rice starch. NARS = Native autoclaved resistant starch. LS 1 NA = Lintnerized starch treated with 1 N HCl. LS 1.5 NA = Lintnerized starch treated with 1.5 N HCl. LS 2 NA = Lintnerized starch treated with 2 N HCl. LA 1 NA = Lintnerized starch treated with 1 N HCl and autoclaved. LA 1.5 NA = Lintnerized starch treated with 1.5 N HCl and autoclaved. LA 2 NA = Lintnerized starch treated with 2 N HCl and autoclaved. NRS - FT = Native rice starch-Freeze thawed.

**Table 3:** Antioxidant properties of the RD 31 rice flour, native starch, and modified starches.

Type of Starch	TPC (mg GAE/100 g Sample)	DPPH (mg Trolox/100 g Sample)	FRAP (FeSO_4_/100 g Sample)
RF	100.03 ± 1.92 ^a^	38.75 ± 0.04 ^a^	192.17 ± 0.54 ^a^
NRS	95.63 ± 1.40 ^b^	29.95 ± 0.41 ^b^	88.66 ± 0.97 ^b^
LS 1 NA	51.00 ± 0.96 ^d^	27.35 ± 0.23 ^c^	53.85 ± 3.19 ^cd^
LS 1.5 NA	45.41 ± 2.09 ^e^	27.37 ± 0.05 ^c^	51.50 ± 0.53 ^d^
LS 2 NA	43.84 ± 0.90 ^e^	26.43 ± 0.45 ^d^	21.62 ± 1.39 ^ef^
NARS	56.44 ± 0.92 ^c^	24.31 ± 0.16 ^e^	56.25 ± 1.39 ^c^
LA 1 NA	36.19 ± 0.42 ^f^	24.59 ± 0.26 ^e^	22.51 ± 0.80 ^e^
LA 1.5 NA	36.52 ± 0.69 ^f^	24.28 ± 0.22 ^e^	19.34 ± 2.12 ^fg^
LA 2 NA	35.07 ± 1.00 ^f^	23.48 ± 0.38 ^f^	16.54 ± 0.35 ^g^
NRS - FT	60.02±1.17 ^c^	27.77 ± 0.06 ^c^	56.26 ± 0.39 ^c^

Results expressed as mean of triplicate determinations ± standard deviation. Different superscripts within one column denote statistically significant differences (*p* <0.05). TPC, total phenolic content; DPPH, 2,2 diphenyl-1-picrylhydrazyl radical scavenging assay; FRAP, Ferric ion reducing antioxidant power assay; RF = Rice flour. NRS = Native rice starch. NARS = Native autoclaved resistant starch. LS 1 NA = Lintnerized starch treated with 1 N HCl. LS 1.5 NA = Lintnerized starch treated with 1.5 N HCl. LS 2 NA = Lintnerized starch treated with 2 N HCl. LA 1 NA = Lintnerized starch treated with 1 N HCl and autoclaved. LA 1.5 NA = Lintnerized starch treated with 1.5 N HCl and autoclaved. LA 2 NA = Lintnerized starch treated with 2 N HCl and autoclaved. NRS - FT = Native rice starch-Freeze thawed.

**Table 4:** Viscosity properties of the RD 31 rice flour, native starch, and modified starches.

Type of Starch	Viscosity					
	Peak 1	Trough 1	Breakdown	Final Viscosity	Setback	Peak Time
RF	142.00 ± 0.00 ^a^	96.50 ± 0.71 ^a^	45.00 ± 0.00 ^b^	308.50 ± 2.92 ^a^	212.00 ± 2.82 ^a^	5.60 ± 0.00 ^b^
NRS	133.50 ± 2.12 ^b^	72.00 ± 0.00 ^c^	67.50 ± 6.50 ^a^	217.00 ± 0.00 ^b^	145.50 ± 0.71 ^b^	4.90 ± 0.04 ^cd^
LS 1 NA	25.79 ± 0.06 ^d^	11.92 ± 0.47 ^d^	13.88 ± 0.42 ^c^	16.21 ± 0.41 ^d^	4.29 ± 0.06 ^d^	5.37 ± 0.05 ^bc^
LS 1.5 NA	3.75 ± 0.00 ^f^	1.21 ± 0.06 ^f^	2.54 ± 0.06 ^cd^	4.58 ± 0.00 ^f^	3.38 ± 0.06 ^de^	4.73 ± 0.00 ^d^
LS 2 NA	2.54 ± 0.04 ^fg^	1.38 ± 0.06 ^f^	1.17 ± 0.12 ^d^	4.38 ± 0.06 ^f^	3.00 ± 0.00 ^de^	4.90 ± 0.04 ^cd^
NARS	8.00 ± 0.11 ^e^	8.05 ± 0.18 ^e^	0.04 ± 0.06 ^d^	11.34 ± 0.12 ^e^	3.29 ± 0.06 ^de^	6.77 ± 0.05 ^a^
LA 1 NA	0.79 ± 0.04 ^g^	0.58 ± 0.00 ^f^	0.21 ± 0.06 ^d^	0.84 ± 0.12 ^g^	0.25 ± 0.11 ^e^	1.20 ± 0.10 ^f^
LA 1.5 NA	1.17 ± 0.00 ^fg^	1.04 ± 0.06 ^f^	0.13 ± 0.06 ^d^	1.46 ± 0.06 ^g^	0.42 ± 0.00 ^e^	1.07 ± 0.00 ^f^
LA 2 NA	1.09 ± 0.12 ^fg^	1.04 ± 0.06 ^f^	0.04 ± 0.06 ^d^	1.42 ± 0.12 ^g^	0.38 ± 0.06 ^e^	2.94 ± 0.27 ^e^
NRS - FT	80.71 ± 0.06 ^c^	74.67 ± 0.23 ^b^	6.05 ± 0.18 ^cd^	105.92 ± 0.47 ^c^	31.25 ± 0.71 ^c^	7.00 ± 0.00 ^a^

Results expressed as mean of triplicate determinations ± standard deviation. Different superscripts within one column denote statistically significant differences (*p* < 0.05). RF = Rice flour. NRS = Native rice starch. NARS = Native autoclaved resistant starch. LS 1 NA = Lintnerized starch treated with 1 N HCl. LS 1.5 NA = Lintnerized starch treated with 1.5 N HCl. LS 2 NA = Lintnerized starch treated with 2 N HCl. LA 1 NA = Lintnerized starch treated with 1 N HCl and autoclaved. LA 1.5 NA = Lintnerized starch treated with 1.5 N HCl and autoclaved. LA 2 NA = Lintnerized starch treated with 2 N HCl and autoclaved. NRS - FT = Native rice starch-Freeze thawed.

**Table 5:** Color spectra of the RD31 rice flour, native starch, and modified starches.

Type of Starch	L*	a*	b*	Color Intensity
RF	88.68 ± 0.09 ^d^	0.38 ± 0.03 ^e^	9.02 ± 0.04 ^d^	9.03 ± 0.04 ^d^
NRS	92.87 ± 0.17 ^a^	−0.20 ± 0.15 ^f^	4.38 ± 0.31 ^g^	4.39 ± 0.31 ^g^
LS 1 NA	91.13 ± 0.01 ^bc^	1.28 ± 0.02 ^d^	5.75 ± 0.01 ^f^	5.88 ± 0.12 ^f^
LS 1.5 NA	90.61 ± 0.31 ^bc^	1.41 ± 0.07 ^d^	7.21 ± 0.21 ^e^	7.35 ± 0.22 ^e^
LS 2 NA	90.13 ± 0.02 ^cd^	1.49 ± 0.03 ^d^	6.55 ± 0.03 ^ef^	6.71 ± 0.02 ^ef^
NARS	74.01 ± 1.04 ^f^	4.49 ± 0.28 ^b^	19.51 ± 0.91 ^a^	20.38 ± 0.9 ^a^
LA 1 NA	80.52 ± 0.03 ^e^	2.63 ± 0.03 ^c^	15.06 ± 0.05 ^c^	15.29 ± 0.06 ^c^
LA 1.5 NA	72.32 ± 0.39 ^g^	5.15 ± 0.04 ^a^	17.91 ± 0.16 ^b^	18.64 ± 0.14 ^b^
LA 2 NA	75.33 ± 0.23 ^f^	4.69 ± 0.02 ^b^	19.68 ± 0.14 ^a^	20.23 ± 0.23 ^a^
NRS - FT	91.99 ± 1.40 ^ab^	0.07 ± 0.01 ^f^	6.05 ± 0.16 ^fg^	6.05 ± 0.16 ^f^

Results expressed as mean of triplicate determinations ± standard deviation. Different superscripts within one column denote statistically significant differences (*p* < 0.05). L* = lightness. a* = redness/greenness. b* = yellowness/blueness. RF = Rice flour. NRS = Native rice starch. NARS = Native autoclaved resistant starch. LS 1 NA = Lintnerized starch treated with 1 N HCl. LS 1.5 NA = Lintnerized starch treated with 1.5 N HCl. LS 2 NA = Lintnerized starch treated with 2 N HCl. LA 1 NA = Lintnerized starch treated with 1 N HCl and autoclaved. LA 1.5 NA = Lintnerized starch treated with 1.5 N HCl and autoclaved. LA 2 NA = Lintnerized starch treated with 2 N HCl and autoclaved. NRS - FT = Native rice starch: freeze thawed.
